# Satisfaction with Teleophthalmology Services: Insights from Remote Areas of Taiwan

**DOI:** 10.3390/healthcare12080818

**Published:** 2024-04-11

**Authors:** Nancy Chen, Jen-Hung Wang, Cheng-Jen Chiu

**Affiliations:** 1 Department of Ophthalmology, Hualien Tzu Chi Hospital, Buddhist Tzu Chi Medical Foundation, Hualien 970, Taiwan; nancy_chen@tzuchi.com.tw; 2 Department of Medical Research, Hualien Tzu Chi Hospital, Buddhist Tzu Chi Medical Foundation, Hualien 970, Taiwan; paulwang@tzuchi.com.tw; 3 Department of Ophthalmology and Visual Science, Tzu Chi University, Hualien 970, Taiwan

**Keywords:** telemedicine, videoconferencing, teleophthalmology, satisfaction, remote area

## Abstract

During and after the COVID-19 pandemic, teleophthalmology provided access to eye care for rural populations. This study aimed to assess the efficacy of and satisfaction with an integrated real-time videoconferencing module. This project incorporated ophthalmic instruments and telecommunication devices and provided on-site consultations via videoconferencing. Both patients and healthcare providers completed satisfaction questionnaires. From May 2020 to May 2021, this project provided eye care services to 395 patients (aged 6–90 years). The most frequent eye condition was chronic conjunctivitis (n = 197), followed by senile cataract (n = 163), dry eye (n = 103), and refractive error (n = 95). Among them, 40 (10.1%) patients were referred to secondary or tertiary hospitals for further evaluation or treatment. In total, 181 recruited respondents provided good satisfaction scores in all dimensions, including quality of medical care (4.50 of 5.00), financial aspects of care (4.48), supportive attitude toward the project (4.47), quality of service (4.40), and quality of telecommunication (4.40). Women had a substantially more supportive attitude toward the project, and 25 healthcare providers provided low ratings in areas representing the quality of telecommunication (4.04) and user-friendliness of the instrument (4.00). This teleophthalmology system provided efficient and satisfactory eye care to participants in remote communities. However, better internet access and training in instrument use can reduce obstacles to the future implementation of the project.

## 1. Introduction

Telemedicine is an emerging paradigm aiming to diminish geographical barriers and disparities in access to medical care resources [1]. During the COVID-19 pandemic, telehealth achieved more acceptance and popularity in various medical fields, as it bridged the gap in patient care, particularly with regard to inconvenient medical visits in rural and urban areas [2,3]. Arguments against the implementation of telemedicine consider that virtual communication is not comparable with in-person physical examinations [1,4]. This perception is even stronger in the field of ophthalmology [5]. Despite these uncertainties, teleophthalmology has been explored as a possible solution for rural and remote areas.

**Figure 1:**
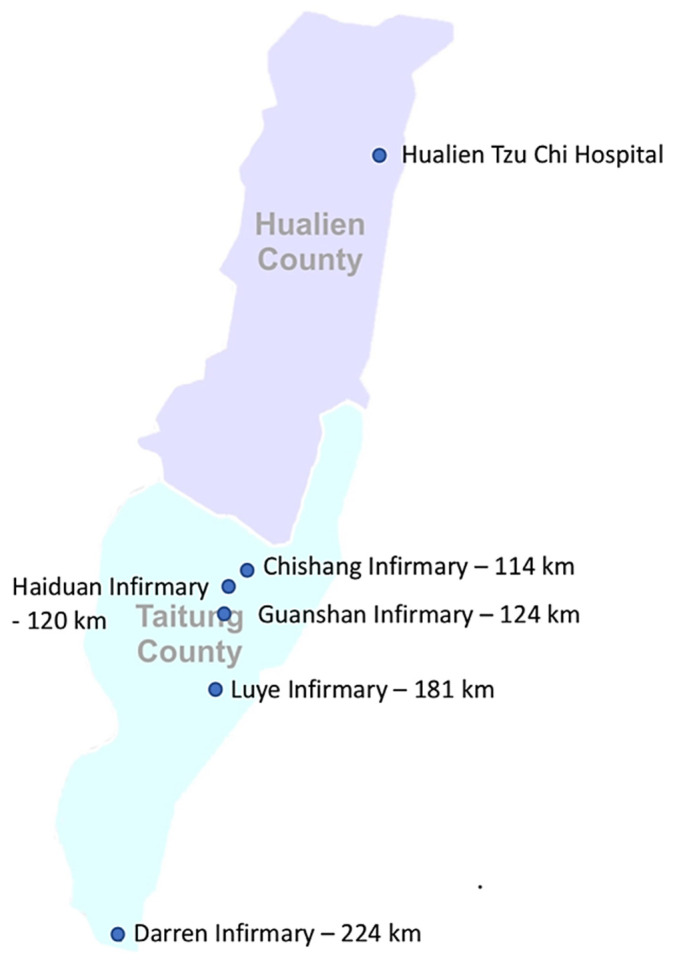
Map of eastern Taiwan showing the involved infirmaries and the distance between these infirmaries and the medical center, Hualien Tzu Chi Hospital.

The terrain in eastern Taiwan where indigenous tribes and rural villages are scattered is long, narrow, and mountainous. Considering the large distance between the consulting medical center and associated remote infirmaries, access to specialized medical care, such as eye care, is limited. The nearest township in this study was located 114 km from Hualien Tzu Chi Hospital and the most distant township was Darren, located 224 km away (Figure 1). Among them, residents of Chishang and Guanshan could access urban areas via express train. On the other hand, the ones in other townships would require several different vehicles to reach the urban area for medical attention. For accurate and timely evaluation while setting up the current project, the Public Health Bureau of the Taitung County Government provided ophthalmic instruments and telecommunication devices for local infirmaries. Although most previous studies have focused on the advances in ophthalmic instruments and broadband internet access that have rapidly facilitated the evolution of teleophthalmology [6,7], relatively few studies have addressed the Implementation of teleophthalmology from a patient perspective [8,9], and even fewer studies have surveyed the satisfaction level of healthcare providers practicing telemedicine [10,11]. This study evaluated the patient’s and health provider’s satisfaction with the application of synchronous videoconference teleophthalmology to determine its effectiveness and identify potential issues for its future implementation.

## 2. Materials and Methods

### 2.1. Setting and Participants

Our virtual vision module (VVM) was equipped with ophthalmic instruments, including an auto kerato-refractometer, tonometer, digital ophthalmoscope, digital slit lamp, computers for data storage and transport, and two iPads for videoconferencing. Videoconferencing was performed using software developed by FarEasTone Telecommunications. All telehealth applications, supported by a continuous 80 MHz bandwidth 5G network, were designed to ensure that the remote diagnosis and treatment platform fulfilled the need for privacy protection, medical image transformation, and seamless interactive communications. Images and data were transferred to and stored in our medical center system. The VVM team included 1 general practitioner, 2 to 3 public health nurses from each local infirmary, ophthalmologists, and administrative personnel from the medical center. Ethical approval was obtained from the Institutional Review Board of the Hualien Tzu Chi General Hospital (IRB109-198-B), which waived the requirement for informed consent because the anonymous questionnaire posed a low or negligible risk to the participants.

**Figure 2:**
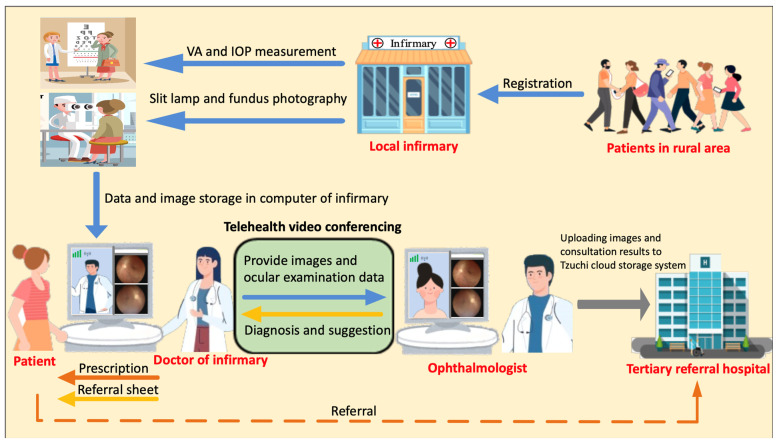
Flowchart showing the virtual vision module process.

The VVM team members of the infirmaries were trained to use the ophthalmic devices and teleophthalmology equipment for 3 months. They collected demographic data, general health information, and eye condition data according to a standardized chart. Patients underwent a comprehensive ophthalmic examination, including visual acuity determination, noncontact tonometry, slit lamp biomicroscopy, and fundoscopy. The ocular examination results, including visual acuity and intraocular pressure, were recorded using a teleophthalmology chart. This chart was photographed and transferred to the videoconferencing platform along with the images acquired by infirmary members using a digital slit lamp and fundoscope. Next, the general practitioner from the infirmary conducted a synchronous video consultation with the ophthalmologist, during which the ophthalmologist input all information collected by the infirmary member into the electronic medical records (EMR). Both images and EMR were subsequently transferred to and stored in the medical center system. After a real-time interview with the patient, the ophthalmologist made a diagnosis and indicated the corresponding treatment, which was administered to the patient at the infirmary. The VVM process is illustrated in detail in Figure 2. The definition of ocular diseases and further details on ocular examinations are available elsewhere [12,13].

When a referral was warranted based on our prior research [14], the public health nurses or infirmary coordinators would arrange appointments at secondary or tertiary hospitals and furnish the necessary referral documents. Patients referred in this manner were eligible to waive their copayment upon presenting the required referral documents.

### 2.2. Questionnaire Development

The satisfaction survey questionnaires for patients and healthcare providers were developed using a rational methodology [15]. Seven professionals, comprising three physicians and four public health experts, developed the questionnaires based on their expertise in their respective knowledge domains and prior research [9,16,17]. The patient satisfaction survey comprised 15 questions covering 5 key areas: financial aspects of care, quality of medical care, quality of service, supportive attitude toward the project, and quality of telecommunication. Similarly, the healthcare provider satisfaction survey consisted of 13 questions spanning the following 5 domains: user-friendliness of the instrument, quality of medical care, quality of service, supportive attitude toward the project, and quality of telecommunication. Both surveys employed a 5-point Likert scale, with scores ranging from 1 (strongly disagree) to 5 (strongly agree). Patient satisfaction data were collected using convenience sampling through on-site or telephone interviews. This method was conducted based on the accessibility and availability of project participants. Patients with literacy and normal cognitive abilities were approached by a research assistant to ascertain their willingness to participate in a questionnaire.

A Cronbach alpha was used to evaluate the reliability of the responses. The reliability of the responses in the five areas of satisfaction survey questionnaires was 0.88, 0.77, 0.70, 0.87, and 0.75 for patients and 0.90, 0.82, 0.97, 0.80, and 0.77 for healthcare providers, respectively. The scale-level content validity index (S-CVI) was assessed using the viewpoints of a panel of experts comprising seven raters. The S-CVIs of satisfaction survey questionnaires for patients and healthcare providers were 0.92 and 0.95, respectively.

### 2.3. Statistical Analysis

The participants’ baseline characteristics were expressed as frequencies, proportions, or means ± standard deviations, depending on the characteristics of each variable. The independent *t*-test was used to compare the means of continuous variables, including age and satisfaction score, between different groups. The chi-square test or Fisher’s exact test was used to evaluate the association between two categorical variables, including age group, time of visit, referral (yes/no), education level, comorbidities (yes/no), occupation, seniority, and experience of telecommunication. *p*-values of <0.05 were considered to indicate statistical significance. All statistical analyses were performed using SPSS software version 17.0 (SPSS Inc., Chicago, IL, USA).

## 3. Results

### 3.1. Demographic Characteristics of Participants

From 20 May 2020 to 11 May 2021, the VVM project was implemented at five local infirmaries located in five villages in Taitung County, eastern Taiwan (Figure 1). The VVM provided eye care services to 395 patients (mean age, 65.13 ± 15.60 years; range, 6–90 years). Approximately 70% of the patients were aged >60 years, and the male-to-female ratio was 2:1 (265:130; Table 1).

Examination of the frequency of visits revealed interesting patterns within the patient population (Table 1). Most patients (83.5%; 330) had a single visit, suggesting that a significant proportion of patients sought medical attention for a specific concern. Meanwhile, 12.2% (48) of patients returned for a second visit, indicating a need for ongoing or follow-up care. A smaller proportion of patients (3%; 12) had three visits, indicating a more complex medical situation requiring multiple assessments. Remarkably, an extremely small but notable subset constituting 5 patients (1.3%) had >3 visits, suggesting a consistent and prolonged need for medical attention.

### 3.2. Frequency of Eye Conditions and Number of Referrals

Based on the International Classification of Diseases 10th Revision (ICD-10 code) reported in the EMR, the most frequent eye condition was chronic conjunctivitis (n = 197), followed by senile cataract (n = 163), dry eye (n = 103), and refractive error (n = 95). Ocular inflammations, such as conjunctivitis, could be readily improved by eyedrops. Senile cataract patients could be followed up according to their visual acuity and slit lamp photos, while patients with refractive errors were referred to an optometrist based on their daily needs, such as presbyopia impeding reading or far vision impairment affecting driving. 

**Figure 3:**
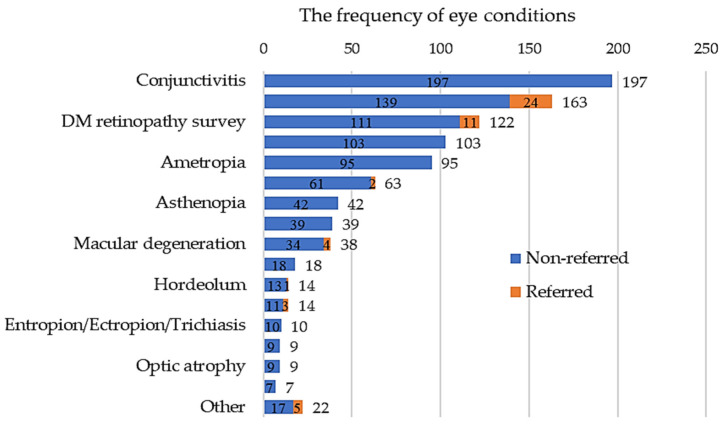
The frequency of eye conditions and the number of referrals.

One hundred and twenty-two visits were recorded for diabetic retinopathy screening (Figure 3). Among them, those suspected of proliferative retinopathy were referred (n = 11), while others were followed up annually or according to the guideline, along with education about diabetic complication prevention. Forty (10.1%) patients were referred to secondary or tertiary hospitals for further evaluation or treatment. Among the patients referred to our hospital, four underwent surgery for vision-impairing cataracts, while optical coherence tomography (OCT) and fundus angiography (FAG) were performed in four patients with macular degeneration, followed by subsequent treatments. Additionally, three patients with vitreous opacity and two with mild macular puckers were placed under outpatient department follow-up. Two patients underwent irrigation for nasolacrimal duct stenosis, two were prescribed atropine eye drops for myopia, one underwent incision and curettage for a hordeolum, and one was evaluated for an eye contusion. Patients who did not require specialized examinations or treatments were returned to their original infirmary for continued care. Education, suggestions, or medication were provided for those conditions (left side of the entity’s bar in Figure 3) that could be effectively managed in the infirmary.

### 3.3. Demographic Analysis of Questionnaire Respondents

A research assistant conducted 181 on-site or telephone questionnaire interviews, which provided insights into respondents’ demographic characteristics and satisfaction scores, as presented in Table 2. Notably, the age and sex distribution of the respondents closely resembled that of the project participants, with a women-to-men ratio of approximately 2:1 (115 women and 66 men). Additionally, one-third of the female participants were found to be illiterate. The prevailing comorbidities among the respondents included conditions such as hypertension, diabetes, and coronary heart disease.

### 3.4. Patient Satisfaction

**Figure 4:**
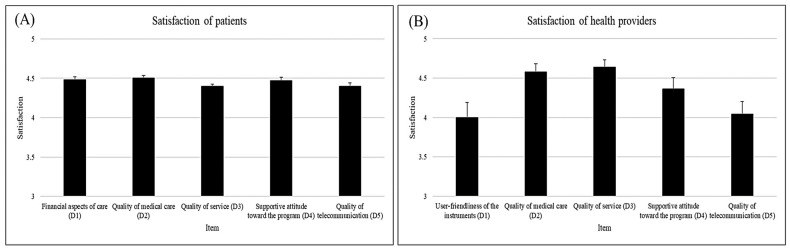
Satisfaction scores of (**A**) patients and (**B**) healthcare providers.

As shown in Table 2 and Figure 4A, the satisfaction survey scores of patients in all five areas were high. The scores were 4.50 of 5 in the quality of medical care (i.e., appropriateness of examination, diagnosis, treatment, and explanation provided by the eye doctor), 4.48 in the financial aspects of care (i.e., time and traffic expense saved), 4.47 in the supportive attitude toward the project (i.e., continued use and willingness to recommend the service), 4.40 in the quality of service (i.e., friendliness and enthusiasm of the healthcare providers and the assistance they provided), and 4.40 in the quality of telecommunication. Women were substantially more supportive of the project (Table 2). We divided the participants into two subgroups to evaluate the study’s external validity. Group 1 included participants from Chishang and Guanshan, and group 2 comprised Haiduan, Luye, and Darren participants. The results showed that the satisfaction scores of the two groups were comparable (Table S3).

### 3.5. Satisfaction of Healthcare Providers

The satisfaction survey scores of healthcare providers were good in all five areas (Table 3, Figure 4B). The scores were 4.58 of 5 in the quality of medical care (i.e., diagnosis, treatment, and explanations provided by the eye doctor), 4.46 in the quality of service (i.e., referral arrangements and enthusiasm of the eye doctor), 4.36 in the supportive attitude toward the project, 4.04 in the quality of telecommunication, and 4.00 regarding user-friendliness of the instrument. Nevertheless, the areas representing telecommunication quality (score, 4.04) and user-friendliness of instruments (score, 4.00) were slightly low among healthcare providers. We divided the health providers into two subgroups to evaluate the study’s external validity. Group 1 included healthcare providers from Chishang and Guanshan, and group 2 comprised Haiduan, Luye, and Darren healthcare providers. The results showed that the satisfaction scores of the two groups were comparable (Table S4). When comparing the satisfaction scores of patients and healthcare providers, we observed significant differences in two areas: The quality of service was rated lower in the patient group, whereas the quality of telecommunication was rated lower among healthcare providers (Table 4).

## 4. Discussion

In the context of telemedicine, teleophthalmology, which integrates eye care services into remote healthcare delivery, can be categorized based on its specific applications. These applications encompass various functions, such as disease screening (e.g., fundus assessments for diabetic retinopathy) [18,19], follow-up assessments (e.g., monitoring intraocular pressure in patients with glaucoma and using OCT for age-related macular disease and diabetic macular edema), and triaging of new ocular symptoms [20,21,22]. Additionally, when ophthalmic consultation is required, it can be provided asynchronously through store-and-forward or synchronously through real-time interactions [23]. In teleophthalmology, integrating emerging technologies is reshaping the landscape of remote eye examinations. In circumstances of ample budget, advanced imaging modalities like OCT, OCT angiography, visual field analyzers, electrophysiologic testing devices, and the advent of non-invasive hybrid ultrasound stimulation of the visual cortex [24] could have significantly enriched the diagnostic armamentarium available to ophthalmologists. 

This study implemented a synchronous videoconferencing platform (VVM) that serves multiple functions, including screening, triage, referral, and follow-up, all within a single integrated system. In this study, a general practitioner from the local infirmary accompanied the patients during videoconferences. The general practitioner performed an initial evaluation of the patients, summarized their underlying medical conditions, and clarified the primary concerns related to their ocular issues. Therefore, this module differs from other telemedicine programs as it facilitates communication between recipients (patient and general practitioner) and the provider (ophthalmologist). This integrated approach ensures a comprehensive and efficient delivery of eye care services, thus benefiting patients and healthcare providers.

In comparing face-to-face synchronous videoconferencing teleophthalmology with store-and-forward teleophthalmology (Table 5), several vital considerations arise regarding their advantages and limitations. Face-to-face synchronous videoconferencing allows real-time interaction between the patient and the healthcare provider, facilitating immediate feedback and discussion of findings. This synchronous approach is particularly beneficial for urgent cases requiring prompt assessment and management. However, it may be limited by the need for both parties to be available simultaneously, potentially leading to scheduling challenges. On the other hand, store-and-forward teleophthalmology involves capturing images or data at one location and sending them to another location for later review by a specialist. This asynchronous approach offers flexibility in terms of timing, as experts can review the images at a convenient time. However, store-and-forward teleophthalmology lacks the immediate feedback and interaction provided by synchronous videoconferencing, which may be crucial for specific clinical scenarios.

During the unprecedented challenges posed by the COVID-19 pandemic, Patel et al. documented various prevalent symptoms observed during teleophthalmology triage visits, encompassing issues such as conjunctivitis, chalazion, scleritis, visual disturbances, and dry eye [20]. Our own experiences within the VVM practice mirrored these findings, where we frequently encountered ICD-10 codes associated with conjunctivitis, senile cataracts, dry eyes, and refractive error. The majority of these conditions demonstrated effective evaluability through teleophthalmology. Our VVM practice notably included 122 visits specifically for diabetic retinopathy screening. A noteworthy study comparing diagnostic accuracy between technology-based eye care services (TECS) and face-to-face (FTF) examinations revealed substantial agreement in the diagnosis of cataracts and diabetic retinopathy [25].

As highlighted in a prior review by Saleem et al. [6], certain subspecialties, including oculoplastics, neuro-ophthalmology, and pediatrics, may be well-suited for telemedicine due to the external nature of many of their examinations. Conversely, addressing anterior segment pathology, such as keratitis and uveitis, through teleophthalmology presents more challenges. Meanwhile, teleophthalmology has demonstrated utility in medicine reconciliation and the assessment of glaucoma medication tolerance. Additionally, the utilization of fundus photography for diabetic retinopathy screening has proven to be both viable and cost-effective in the view of public health [18,26]. A review proposed that the integration of telehealth with in-person care, particularly post-diagnosis, could be valuable in managing chronic conditions [1]. In summary, teleophthalmology in our VVM setting is suitable for managing stable conditions like monitoring glaucoma intraocular pressure, conducting annual fundoscopy screenings for diabetic patients, and addressing mild ocular symptoms such as itchiness, wateriness, dryness, and soreness of the eye. However, acute deteriorations in vision, elevated intraocular pressure, sudden onset diplopia, suspicion of eyelid tumor, eyeball trauma, and intractable eye pain that does not improve after eyedrops should not be managed remotely through teleophthalmology; instead, immediate referral is recommended.

Although higher health literacy and regular internet access have traditionally been associated with patients’ interest in using telemedicine in rural areas [2], we found that one-third of the female participants were illiterate. This unexpected finding can be attributed to the dedication and rapport established by the patient-end infirmary staff, who were intimately familiar with the village inhabitants and had earned their trust [23].

Notably, we had more female participants who exhibited a more supportive attitude toward our VVM services. This suggests that the utilization of and satisfaction with our VVM teleophthalmology system were not impeded by factors such as lower educational attainment, gender (female), or older age [27,28]. This highlights that our teleophthalmology system was easily accessible and well-received by the remote population, even by those who may traditionally face barriers to technological advancements and healthcare access.

In addition to the well-established efficacy of telemedicine adaptation owing to increased accessibility of eye care services [29], it is important to recognize that the success of this system depends on the acceptance and robust support from the community [4,23]. Herein, we systematically collected satisfaction data from patients and healthcare providers through a comprehensive questionnaire that delved into various aspects of their telemedicine experience.

Previous research has revealed that high satisfaction levels are closely associated with several factors, including patients’ older age, familiarity with the patient-end clinician, interface usability, and appreciation for time and cost savings [9,11,30]. In alignment with these findings, our patients expressed the highest satisfaction levels with the quality of medical care, which assessed the appropriateness of examination, diagnosis, treatment, and explanation provided by the eye doctor. This was closely followed by the financial aspects of care, where patients valued the time and cost savings associated with the teleophthalmology service. Additionally, a supportive attitude toward the project, indicating continued use and willingness to recommend the service, ranked high among satisfaction factors.

Notably, considering that a substantial proportion of our patients were older individuals, on-site videoconferencing consultations, which seamlessly combine examination and treatment in a single session, proved to be a highly convenient and cost-effective approach. This contributed to the high level of satisfaction among our patients, thus underlining the effectiveness of this model in catering to the unique needs of the older population.

In our study, infirmary healthcare providers expressed confidence in the quality of medical care (4.58) and quality of service (4.64). However, they provided slightly low ratings for the quality of telecommunication (4.04) and user-friendliness of instruments (4.0). These findings align with concerns from other studies regarding incomplete assessments using telehealth [10,11] and the need for training and support for using the platform [4,10]. Healthcare providers encountered issues with unstable voice and video quality during real-time videoconferencing. They also faced difficulties transferring patient data from examination instruments to the conferencing device. Some challenges arose in conducting examinations on some patients; for instance, elderly patients might not comply with placing their face on the chin-rest, leading to difficulties in obtaining a clear image of a slit lamp or fundoscopy. Improving internet infrastructure could enhance the quality of telecommunication. Furthermore, on-the-job training and seminars with specialists are essential to mastering the technique comprehensively.

We observed remarkable differences in satisfaction scores between patients and healthcare providers, particularly in the areas of quality of service and quality of telecommunication. From a service quality perspective, infirmary healthcare providers assessed the referral process and the friendliness and enthusiasm of the consulting ophthalmologist. Conversely, patients evaluated the teleophthalmology service as a more complex entity, encompassing aspects such as appointment procedures, referrals, and communication skills of both infirmary healthcare providers and those at the medical center. Therefore, achieving high satisfaction scores in the patients’ survey proved to be more challenging.

In this study, healthcare providers, comprising doctors and nurses from local infirmaries, assumed dual responsibilities by serving as service providers for patients and service recipients during interactions with the consulting ophthalmologist. Importantly, these healthcare providers were not employed by the project administration and were not involved in the program’s design. This distinctive dual role distinguishes them from participants in conventional satisfaction surveys, where the medical staff generally function solely as care providers. Thus, the scores of such participants hold unique significance [11,17]. Similar to previous studies, the quality of telecommunication was rated high by patients [11,17]. In teleophthalmology, the medical staff bears the responsibility for the preparatory process, ensuring appropriate device setup, whereas patients utilize the equipment once it is ready. The survey findings highlight the importance of enhancing both internet and information technology infrastructures and the development of training programs for the medical staff. These steps are vital to ensure the safe and effective operation of teleophthalmology in the future, aligning with previous research [20,31,32].

Furthermore, although synchronous videoconferencing is a cost-effective approach from the perspective of patients involved in telemedicine [17], pre-consultation preparations place a relatively time-consuming burden on healthcare personnel [20]. Overcoming this challenge necessitates increased manpower support and appropriate reimbursement to streamline and facilitate the process.

To assess the study’s external validity, we stratified the five townships into two subgroups based on traffic convenience. Our subgroup analysis also revealed consistent satisfaction scores across all domains for both participants and healthcare providers. This suggests that our VVM model is adaptable across varied populations, ensuring equivalent satisfaction and reinforcing its external validity. 

In summary, in our VVM model, teleophthalmology offers significant advantages, including enhanced accessibility to eye care services, improved compliance via infirmaries, and patient-end support to overcome technological barriers. However, notable disadvantages include the high cost of devices, the requirement for substantial training for program setup, and the time-consuming nature of pre-consultation examinations. Common applications of teleophthalmology include vision check-ups, monitoring of intraocular pressure for patients with glaucoma, screening for diabetic retinopathy, and triaging of new-onset eye conditions. Despite these advantages, teleophthalmology has certain limitations, such as the inability to conduct peripheral fundoscopy, provide immediate surgical treatment, and perform comprehensive surveys of patients suspected of glaucoma, age-related macular diseases, or optic neuropathy. In such cases, referrals to secondary or tertiary hospitals become necessary.

There are several limitations in this study. First, our sample size was relatively small; thus, potential bias may exist in patient selection. Nevertheless, the age and sex distribution of respondents closely resembled that of project participants, thus mitigating potential bias. Additionally, the findings of our study may not be universally applicable due to variations in technology and infrastructure. Furthermore, not all referred patients visited the consulting medical centers, resulting in some unknown final diagnoses. In retrospect, we understand that involving an independent panel of international experts could have offered a more objective evaluation. However, due to the project’s initiation during the pandemic, these participants were the most viable subjects available without any associated risks. However, they may lack the professional expertise to comprehensively assess the medical system, and their opinions may not align with clinical outcomes. Further research is warranted to overcome these limitations and gain a more comprehensive understanding of the topic.

## 5. Conclusions

The survey results revealed high satisfaction levels with teleophthalmology services among rural participants and healthcare providers. For the successful future implementation of teleophthalmology, it is crucial to improve telecommunication infrastructure and offer training programs for instrument use. With the ongoing expansion of teleophthalmology during and after the COVID-19 pandemic, VVM videoconferencing has emerged as an efficient alternative for delivering eye care services.

## Figures and Tables

**Table 1:** Patient characteristics.

Variables	Male	Female	Total	*p*-Value
N	130	265	395	
Age (y/o) ^a^	65.55 ± 15.90	64.93 ± 15.48	65.13 ± 15.60	0.713
Age group, n (%) ^b^				0.714
≤60 y/o	33 (25.4)	80 (30.2)	113 (28.6)	
61–70 y/o	41 (31.5)	72 (27.2)	113 (28.6)	
71–80 y/o	37 (28.5)	77 (29.1)	114 (28.9)	
>80 y/o	19 (14.6)	36 (13.6)	55 (13.9)	
Number of visits, n (%) ^b^				0.283
1	111 (85.4)	219 (82.6)	330 (83.5)	
2	17 (13.1)	31 (11.7)	48 (12.2)	
3	1 (0.8)	11 (4.2)	12 (3.0)	
>3	1 (0.8)	4 (1.5)	5 (1.3)	
Referral, n (%) ^b^	20 (15.4)	20 (7.5)	40 (10.1)	0.015 *

Data are presented as n (%) or mean ± standard deviation; * *p*-values of <0.05 were considered to indicate statistical significance; ^a^: independent *t*-test; ^b^: chi-square test or Fisher’s exact test; y/o: year old.

**Table 2:** Satisfaction survey of patients.

Variables	Male	Female	Total	*p*-Value
N	66	115	181	
Age group, n (%) ^b^				0.719
≤60 y/o	13 (19.7)	31 (27.0)	44 (24.3)	
61–70 y/o	21 (31.8)	31 (27.0)	52 (28.7)	
71–80 y/o	20 (30.3)	32 (27.8)	52 (28.7)	
>80 y/o	12 (18.2)	21 (18.3)	33 (18.2)	
Education, n (%) ^b^				<0.001 *
Illiterate	2 (3.0)	36 (31.3)	38 (21.0)	
Primary school	28 (42.4)	44 (38.3)	72 (39.8)	
Junior high school	16 (24.2)	4 (3.5)	20 (11.0)	
Senior high school	13 (19.7)	19 (16.5)	32 (17.7)	
Above college	7 (10.6)	12 (10.4)	19 (10.5)	
Comorbidities, n (%) ^b^				
DM (%)	25 (37.9)	28 (24.3)	53 (29.3)	0.054
HTN (%)	30 (45.5)	46 (40.0)	76 (42.0)	0.474
Coronary heart disease (%)	19 (28.8)	22 (19.1)	41 (22.7)	0.135
Satisfaction (maximum score is 5) ^a^				
Financial aspects of care (D1)	4.44 ± 0.62	4.5 ± 0.53	4.48 ± 0.56	0.457
Quality of medical care (D2)	4.45 ± 0.46	4.53 ± 0.51	4.50 ± 0.49	0.323
Quality of service (D3)	4.42 ± 0.37	4.38 ± 0.36	4.40 ± 0.37	0.486
Supportive attitude toward the project (D4)	4.31 ± 0.69	4.56 ± 0.56	4.47 ± 0.62	0.009 *
Quality of telecommunication (D5)	4.42 ± 0.56	4.39 ± 0.54	4.40 ± 0.55	0.726

DM, diabetes mellitus; HTN, hypertension; data are presented as n (%) or mean ± standard deviation; * *p*-values of <0.05 were considered to indicate statistical significance; ^a^: independent *t*-test ; ^b^: chi-square test or Fisher’s exact test; y/o: year old.

**Table 3:** Satisfaction survey of healthcare providers.

Variables	Male	Female	Total	*p*-Value
N	2	23	25	
Age group, n (%) ^b^				0.597
21–30 y/o	0 (0.0)	2 (8.7)	2 (8.0)	
31–40 y/o	0 (0.0)	11 (47.8)	11 (44.0)	
41–50 y/o	2 (100.0)	9 (39.1)	11 (44.0)	
51–60 y/o	0 (0.0)	1 (4.3)	1 (4.0)	
Occupation, n (%) ^b^				0.010 *
Doctor	2 (100.0)	1 (4.3)	3 (12.0)	
Nurse	0 (0.0)	22 (95.7)	22 (88.0)	
Seniority, n (%) ^b^				0.680
1–3 years	0 (0.0)	1 (4.3)	1 (4.0)	
4–6 years	0 (0.0)	4 (17.4)	4 (16.0)	
7–10 years	0 (0.0)	8 (34.8)	8 (32.0)	
>10 years	2 (100.0)	10 (43.5)	12 (48.0)	
Experience of telecommunication, n (%) ^b^				0.487
1–3 months	0 (0.0)	7 (30.4)	7 (28.0)	
3–6 months	0 (0.0)	7 (30.4)	7 (28.0)	
>6 months	2 (100.0)	9 (39.1)	11 (44.0)	
Satisfaction (maximum score is 5) ^a^				
User-friendliness of the instruments (D1)	4.25 ± 1.06	3.98 ± 0.97	4.00 ± 0.96	0.709
Quality of medical care (D2)	5.00 ± 0.00	4.54 ± 0.53	4.58 ± 0.52	0.241
Quality of service (D3)	4.83 ± 0.24	4.62 ± 0.47	4.64 ± 0.46	0.547
Supportive attitude toward the project (D4)	4.50 ± 0.71	4.35 ± 0.76	4.36 ± 0.74	0.788
Quality of telecommunication (D5)	3.75 ± 0.35	4.07 ± 0.83	4.04 ± 0.80	0.605

Data are presented as n (%) or mean ± standard deviation; * *p*-values of <0.05 were considered to indicate statistical significance; ^a^: independent *t*-test; ^b^: chi-square test or Fisher’s exact test; y/o: year old.

**Table 4:** Comparison of satisfaction scores between patients and healthcare providers.

Construct	Patients	Healthcare Providers	*p*-Value
N	181	25	
Quality of medical care ^a^	4.50 ± 0.49	4.58 ± 0.52	0.448
Quality of service ^a^	4.40 ± 0.37	4.64 ± 0.46	0.004 *
Supportive attitude toward the project ^a^	4.47 ± 0.62	4.36 ± 0.74	0.418
Quality of telecommunication ^a^	4.40 ± 0.55	4.04 ± 0.80	0.004 *
Financial aspects of care	4.48 ± 0.56	NA	NA
User-friendliness of the instruments	NA	4.00 ± 0.96	NA

Data are presented as mean ± standard deviation. NA, not available; * *p*-values of <0.05 were considered to indicate statistical significance; ^a^: independent *t*-test.

**Table 5:** Comparison of two teleophthalmology modalities.

Aspects	Synchronous Video Conference	Store-and-Forward
Technological requirement	Real-time conferencing and examination devices, stable internet connection	Imaging instruments for capturing and transforming examination data
Scalability	Requires simultaneous participation of patient and healthcare providers, less scalable	Does not require simultaneous availability, schedule more flexible, more scalable
Patient engagement	Higher due to interaction with the healthcare providers	Less, no direct interaction during image capture
Diagnostic accuracy	High due to immediate feedback and discussion of findings	Moderate, objective review of images, but lack interaction
Cost-effectiveness	Generally high if operate properly	High, requires less equipment and healthcare staff, lower ongoing cost

## Data Availability

Data are contained within the article and Supplementary Materials.

## Supplementary Materials

The following supporting information can be downloaded at: https://www.mdpi.com/article/10.3390/healthcare12080818/s1, Table S1: Satisfaction survey questionnaire for patients; Table S2: Satisfaction survey questionnaire for healthcare providers; Table S3: Comparison of satisfaction scores between two groups of patients; Table S4: Comparison of satisfaction scores between two groups of healthcare providers.
