# Supplementary material for: Satisfaction with Teleophthalmology Services: Insights from Remote Areas of Taiwan
Source: Healthcare (Basel). 2024 Apr 11;12(8):818. doi: 10.3390/healthcare12080818 (PMC11050710; doi:10.3390/healthcare12080818)
Supplement: Supplementary file 1 [file healthcare-12-00818-s001.zip › Table S1. Questionair for satisfaction of patients.pdf]

Table S1. Questionnaire of satisfaction survey for patients.

Question	Construct
Q1. I agree that the teleophthalmology service saved time and the traffic expense for accessing medical care.	D1
Q2. The time for examination and consulting during teleophthalmology service is reasonable.	D1
Q3. The examination of visual acuity, intraocular pressure, and fundus photography provided by the teleophthalmology service can help me understand the conditions of my eyes.	D2
Q4. I am satisfied with the explanation given by the eye care specialist.	D2
Q5. The eye care specialist arranged appropriate appointments and referral services.	D3
Q6.The medical staff treated me in a friendly manner.	D3
Q7. The medical staff was enthusiastic about communicating with the patients.	D3
Q8. I agree that the teleophthalmology service provides me with appropriate medical care.	D2
Q9. Teleophthalmology is beneficial to the treatment of my illness.	D2
Q10. I would continue to utilize teleophthalmology services.	D4
Q11. I would recommend teleophthalmology services to my family and friends.	D4
Q12. I consider the voice and visual quality of the teleophthalmology equipment acceptable.	D5
Q13. I agree that videoconferencing utilization made communicating with the eye care specialist comprehensive.	D3
Q14. I think that teleophthalmology consulting is equivalent to a face-to-face appointment.	D5
Q15. I am satisfied with the overall quality provided by the teleophthalmology service.	D4

D1, Financial aspects of care; D2, Quality of medical care; D3, Quality of service; D4, Supportive attitude toward the project; D5, Quality of telecommunication.
