# Supplementary material for: Satisfaction with Teleophthalmology Services: Insights from Remote Areas of Taiwan
Source: Healthcare (Basel). 2024 Apr 11;12(8):818. doi: 10.3390/healthcare12080818 (PMC11050710; doi:10.3390/healthcare12080818)
Supplement: Supplementary file 1 [file healthcare-12-00818-s001.zip › Table S2. Questionaire for satisfaction of healthecare providers.pdf]

Table S2. Questionnaire of satisfaction survey for healthcare provides.

Question	Construct
Q1. The eye doctor provided professional knowledge.	D2
Q2. The eye doctor explained the condition clearly to the patient.	D2
Q3. The eye doctor explained the usage of the medication to the patient well.	D2
Q4. The eye doctor is enthusiastic about communicating with patients and understanding their condition.	D3
Q5. The eye doctor treated patients in a friendly manner.	D3
Q6. Teleophthalmology benefits patients.	D3
Q7. The addition of teleophthalmology service does not affect the infirmary routine of preparation.	D1
Q8. It is easy to operate the devices and instruments of teleophthalmology.	D1
Q9. The eye doctor provided appropriate referral suggestions.	D3
Q10. The patients are satisfied with the services provided by teleophthalmology.	D4
Q11. I would recommend teleophthalmology services to my family and friends.There was no disruption of videoconferencing (sounds or images) during the teleophthalmology consult.	D5
Q12. The images and sounds provided by teleophthalmology equipment are appropriate.	D4
Q13. Providing teleophthalmology is a good medical policy.	D5

D1, User-friendliness of instruments; D2, Quality of medical care; D3, Quality of service; D4, Supportive attitude toward the project; D5, Quality of telecommunication.
