# Supplementary material for: Satisfaction with Teleophthalmology Services: Insights from Remote Areas of Taiwan
Source: Healthcare (Basel). 2024 Apr 11;12(8):818. doi: 10.3390/healthcare12080818 (PMC11050710; doi:10.3390/healthcare12080818)
Supplement: Supplementary file 1 [file healthcare-12-00818-s001.zip › Table S3.pdf]

Table S3. Comparison of satisfaction scores between two groups of patients.

Variables	Group 1	Group 2	Total	P-value
N	104	77	181	
**Satisfaction (maximum score is 5)[a]**				
Financial aspects of care (D1)	4.44±0.56	4.53±0.56	4.48±0.56	0.289
Quality of medical care (D2)	4.48±0.50	4.53±0.49	4.50±0.49	0.521
Quality of service (D3)	4.42±0.36	4.37±0.37	4.40±0.37	0.416
Supportive attitude toward the program (D4)	4.46±0.64	4.48±0.59	4.47±0.62	0.849
Quality of telecommunication (D5)	4.40±0.55	4.39±0.55	4.40±0.55	0.863

Data are presented as mean ± standard deviation.

*p-value<0.05 was considered statistically significant after test. [a] independent t test

Group 1: Chishang or Guanshan counties; Group 2: Haiduan or Luye or Darren counties
