# Supplementary material for: Satisfaction with Teleophthalmology Services: Insights from Remote Areas of Taiwan
Source: Healthcare (Basel). 2024 Apr 11;12(8):818. doi: 10.3390/healthcare12080818 (PMC11050710; doi:10.3390/healthcare12080818)
Supplement: Supplementary file 1 [file healthcare-12-00818-s001.zip › Table S4.pdf]

Table S4. Comparison of satisfaction scores between two groups of healthcare providers.

Variables	Group 1	Group 2	Total	P-value
N	11	14	25	
**Satisfaction (maximum score is 5)[a]**				
User-friendliness of the instruments (D1)	4.14±0.98	3.89±0.96	4.00±0.96	0.539
Quality of medical care (D2)	4.80±0.33	4.41±0.59	4.58±0.52	0.064
Quality of service (D3)	4.79±0.31	4.52±0.53	4.64±0.46	0.159
Supportive attitude toward the program (D4)	4.68±0.46	4.11±0.84	4.36±0.74	0.053
Quality of telecommunication (D5)	3.91±0.89	4.14±0.74	4.04±0.80	0.481

Data are presented as mean ± standard deviation.

*p-value<0.05 was considered statistically significant after test. a independent t test

Group 1: Chishang or Guanshan counties; Group 2: Haiduan or Luye or Darren counties
